# RURAL OLDER ADULTS’ SOCIAL DISCONNECTION AND DEPRESSION DURING THE PANDEMIC

**DOI:** 10.1093/geroni/igae098.2679

**Published:** 2024-12-31

**Authors:** Jung Sim Jun, Kyoung Hag Lee

**Affiliations:** Kansas State University, Kansas State University, Kansas, United States; Wichita State University, Wichita, Kansas, United States

## Abstract

During the pandemic, 24% of older adults reported anxiety or depression, with higher rates among those with low socioeconomic status and poor health, particularly among female older adults (Koma et al., 2020). Older adults in rural communities face greater challenges due to underlying health conditions and limited resources (Henning-Smith, 2020; Pender et al., 2019). Therefore, this study aimed to examine the association between social isolation, social support, and mental health among this population. The research team recruited 103 rural-dwelling older adults using purposive sampling. Measurements included: the Geriatric Depression Scale-Short Form (Sheikh & Yesavage, 1986), Lubben Social Network Scale-6 (Lubben et al., 2006), and Multidimensional Scale of Perceived Social Support (Zimet et al., 1988). Six socio-demographic variables were used as control variables: age, gender, final education, annual household income, perceived health, and driving capacity. There was a positive association between social isolation and depressive symptoms (B = 0.24, SE = 0.07, p ≤.001; B = 0.20, SE = 0.08, p ≤.01) while social support from significant others was negatively associated with depressive symptoms (B = -0.35, SE = 0.11, p ≤.001). However, social support from family, from friends, and age were not significantly related to depressive symptoms. A lack of social resources is a risk factor for rural older adults, potentially leading to increased mental health issues. Social service workers should focus on improve social support systems among rural older adults to establish social networks and create social resources.

